# An objective measure of insight in hoarding Disorder: Associations with severity, functioning, and neuropsychological performance

**DOI:** 10.1016/j.jpsychires.2025.10.065

**Published:** 2025-10-28

**Authors:** Jessica J. Zakrzewski, Elizabeth W. Twamley, Michael L. Thomas, Catherine R. Ayers

**Affiliations:** a Research Service, VA San Diego Healthcare System, San Diego, CA, USA; b Department of Psychiatry, UC San Diego, La Jolla, CA, USA; c Center of Excellence for Stress and Mental Health, VA San Diego Healthcare System, San Diego, CA, USA; d Department of Psychology, Colorado State University, Fort Collins, CO, USA; e Psychology Service, VA San Diego Healthcare System, San Diego, CA, USA

**Keywords:** Anosognosia, Insight, Veterans, Veterans Healthcare, Hoarding disorder, Clutter, Symptom severity, Functioning, Cognition

## Abstract

Hoarding Disorder (HD) is defined by the inability to discard objects until clutter becomes functionally impairing. A DSM-5 specifier for HD is lack of insight. A recent study found links between insight (using an objective clutter proxy) and inhibitory/cognitive control in HD. We aimed to explore associations between insight (using the same clutter proxy) and symptom severity, functioning, and cognition in Veterans with HD. 122 Veterans seeking treatment for HD completed pre-treatment assessments, including home-based assessments of clutter volume using the Clutter Imaging Rating Scale (CIR), HD severity measures, self-reported functioning, and neuropsychological testing. Insight was defined as the difference between the assessor and self-rating of the CIR (i.e., CIR-error). T-tests and regressions were used to evaluate the relationships between measures. The majority of the Veterans were older (*m* = 62), male (61 %), and White (57 %), with some college education. On clinical interview, only 10 % of the sample were rated with impaired insight. The mean CIR-error score was in the impaired range, with 47 % of the Veterans underreporting clutter. Lower HD severity and higher self-reported functioning were related to lower insight. Neuropsychological test performance was related to insight, but with small effects in varying directions. Nearly half of treatment-seeking Veterans demonstrated impairment in insight into levels of clutter, similar to previous work. Objective insight ratings demonstrated better sensitivity than clinician interviews for insight impairments. Lower insight was related to lower self-reported HD severity and higher self-reported functioning, raising the question of a potential insight paradox in HD.

## 1. Introduction

Anosognosia, or a lack of insight, occurs across a variety of neurological and psychiatric disorders, including dementia, traumatic brain injury, stroke, schizophrenia, and depression, among other conditions (Aleman et al., 2006; Bisiach et al., 1986; Rosen, 2011; Steward and Kretzmer, 2022; Subotnik et al., 2020; Yen et al., 2007). Current literature recognizes impaired insight as a multidimensional construct which can range from lack of awareness of an illness, its symptoms, and the need for treatment as well as recognition of the effect an illness has on your thoughts, moods, and actions (Amador et al., 1993; David, 1990; Van Camp et al., 2017). When anosognosia is present, individuals affected have lower treatment compliance, greater symptom severity, increased likelihood of comorbidities, and generally a worse overall prognosis (Aleman et al., 2006; Bisiach et al., 1986; Rosen, 2011; Steward and Kretzmer, 2022; Subotnik et al., 2020; Yen et al., 2007). Contemporary research exploring the neurobiological underpinnings of anosognosia, especially in psychosis and dementia, has emphasized the role of executive functioning and error-monitoring in disordered awareness (Agnew and Morris, 1998; Aleman et al., 2006; David et al., 2012; Klein et al., 2013).

One of the central challenges in studying anosognosia in psychiatric illness is its measurement. Various clinician-administered and self-report tools aim to assess clinical and cognitive aspects of insight (Amador et al., 1993; Beck, 2004; Beck and Warman, 2004; Eisen et al., 1998). However, insight can vary widely both within and across disorders, many of which involve internalized and subjective symptoms, making objective assessment especially difficult. Hoarding Disorder (HD), however, is unique among psychiatric conditions in that its severity is externally observable through physical clutter. The Diagnostic and Statistical Manual of Mental Disorders, Fifth Edition (DSM-5), defines HD by the accumulation of clutter that causes distress or impairment, and includes an insight specifier due to the frequent presence of impaired awareness in HD (American Psychiatric et al., 2013). Impairments of insight in HD are recognized to occur across aspects of the disorder such as difficulty discarding, excessive acquiring, and clutter (American Psychiatric et al., 2013; Frost et al., 2010). Despite this recognition, the role of insight in HD remains an understudied aspect of its clinical profile.

Prior work examining insight in HD has typically relied on clinician or informant reports, with estimates of impaired insight ranging from 55 to 85 % (Drury et al., 2015; Kim et al., 2001; Matsunaga et al., 2002). However, this work is often plagued by small sample sizes due to challenges in recruiting both individuals with HD and corresponding informants (Drury et al., 2015). Some investigations have relied on interviews with elder service providers, without directly comparing their assessments to client self-reports (Kim et al., 2001). Additionally, self-reported HD severity is often significantly lower than objective ratings with significant variation across studies due to measurement type and the historical conflation of HD with OCD (Bloch et al., 2014; De Berardis et al., 2005; Drury et al., 2015; Matsunaga et al., 2002; Torres et al., 2012). More recent work has examined aspects of impaired insight for specific cognitive processes and found discrepancies between subjective and objective cognitive performance, revealing a dissociation between self-reported and task-based memory functioning (Hartl et al., 2004; Zakrzewski et al., 2022 [Hartl, 2004 #15). Although attentional impairments have been documented in HD (Hallion et al., 2015; Worden and Tolin, 2023), both subjective attentional complaints and objective attentional deficits appear more strongly associated with comorbid anxiety symptoms than with HD (Zakrzewski et al., 2022). Collectively, these findings underscore the methodological challenges in using self-report instruments—whether structured interviews or questionnaires—to reliably assess insight in HD.

A recent study by van Roessel et al. (2022) was the first to develop a truly objective measure of insight in HD. Using the validated picture-based questionnaire, the Clutter Image Rating scale (CIR), participants with clinically diagnosed HD completed the CIR during an in-office assessment. Within one week, trained study personnel conducted in-home assessments using the same instrument. Results indicated that 58 % of participants under-reported the severity of their clutter. Moreover, greater objective clutter levels were associated with more impaired insight. The study also found associations between impaired insight and worse cognitive control (i.e., errors on a Go/No-Go task; increased Stroop task response time). This work is the first of its kind to demonstrate an objective measure of insight and link insight level to cognitive performance in a treatment seeking, community-based sample of individuals with HD. These findings align with prior literature demonstrating associations between impaired insight and deficits in cognitive control in Alzheimer’s disease, OCD, bipolar disorder, and psychotic disorders (Aleman et al., 2006; Amanzio et al., 2011; Camelo et al., 2019; Kashyap et al., 2012).

Objectively measuring insight and its relationship to cognitive functioning is essential not only for understanding the pathophysiology of Hoarding Disorder (HD), but also for advancing the broader construct of insight across psychiatric conditions. Similar to the work by van Roessel et al. (2022), the present study utilized data from individuals enrolled in a clinical trial of behavioral treatment for HD; however, all participants in our sample were Veterans recruited through the VA San Diego Healthcare System (VASDHS). Previous research indicates that Veterans with HD exhibit similar symptom severity and treatment response compared to community samples but experience higher rates of psychiatric and medical comorbidity (Ayers et al., 2018). We examined several aspects of the pre-treatment, at-home study assessment. First, we sought to determine the level of insight and its association with HD severity in this Veteran sample. During this assessment, Veterans self-rated their level of clutter using the CIR while seated in their home, and an independent assessor completed room-by-room ratings on the CIR. Although both ratings occurred during the same visit, we anticipated (1) variability in insight and (2) higher objectively rated clutter would correlate with lower insight. We also examined self-rated disability, functioning, and quality of life scales in association with level of insight. While there is no prior work examining insight and functioning in HD, we anticipated that (3) lower insight would be associated with lower self-reported functioning. Finally, we sought to examine the relationship between insight and cognitive functioning, with the expectation that (4) impaired insight would be associated with impairments in executive functioning.

## 2. Methods

### 2.1. Participants and study procedures

**Fig. 1.**
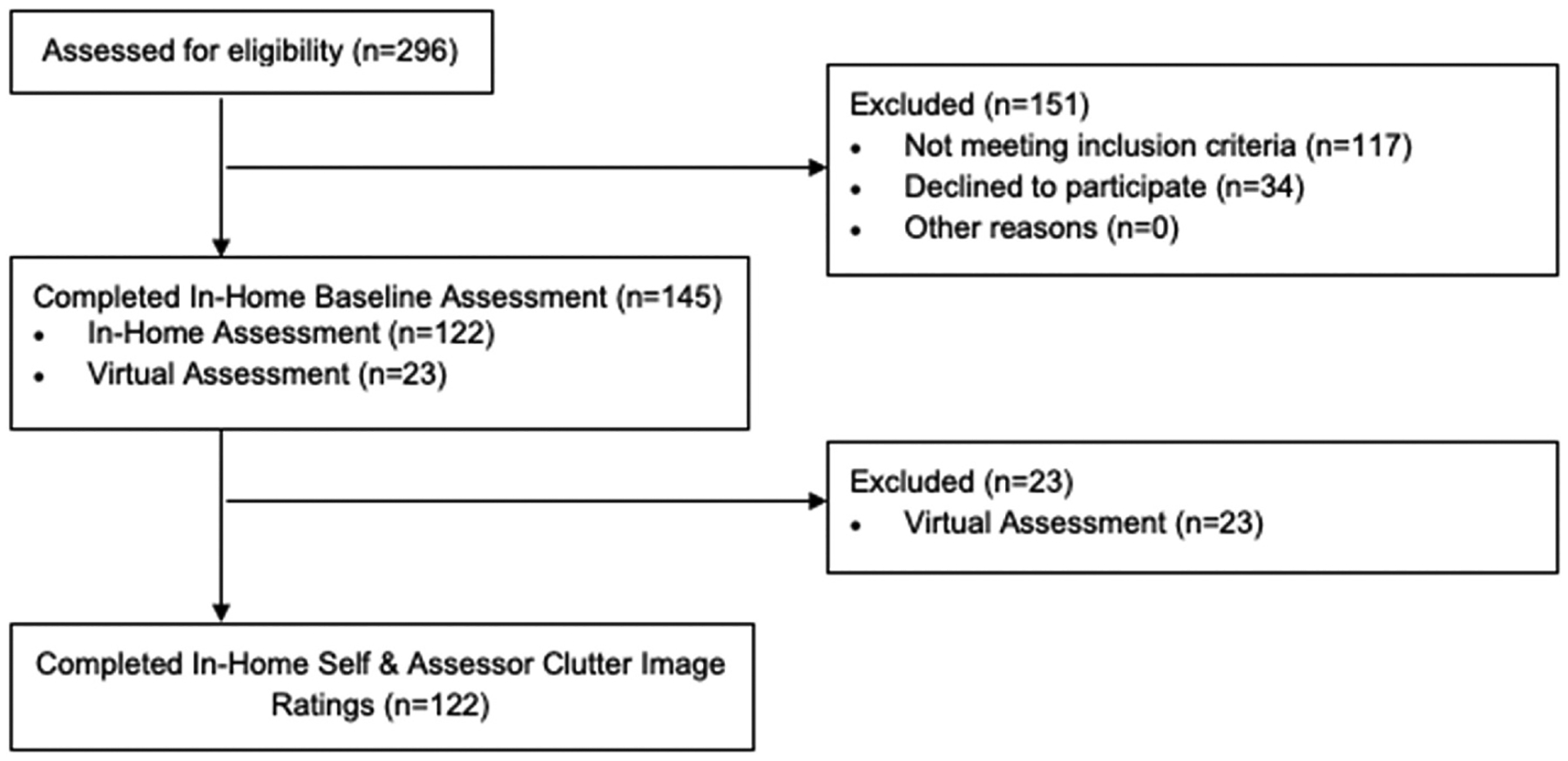
Participant CONSORT diagram.

This study used baseline data collected as part of a randomized controlled trial of behavioral therapy for HD (PI Ayers, VA CSR&D I01CX001149; see Fig. 1 for the CONSORT Diagram). All participants were recruited from the VASDHS from October 2015 through June 2021 via clinical referrals and advertisements. A total of 145 Veterans completed home-based assessments prior to randomization to study treatment arms. Home-based assessments included self-report measures, clinician-rated measures, and neuropsychological testing; 23 of the 145 home-based assessments occurred virtually due to COVID-19 pandemic restrictions; we included data only from the 122 participants who completed home-based assessments in person.

### 2.2. Inclusion/exclusion

All participants were Veterans between the ages of 45–85 who met criteria for HD per the Diagnostic and Statistical Manual of Mental Disorders, Fifth Edition (DSM-5) (American Psychiatric et al., 2013) as measured by the Structured Interview for Hoarding Disorder (SIHD) (Nordsletten et al., 2013). They were required to be stable on medications for at least 12 weeks. Veterans were excluded if they had comorbid diagnoses of a psychotic disorder, bipolar disorder, or substance abuse disorder as measured by the Mini-International Neuropsychiatric Interview (M.I.N.I.) (Sheehan et al., 1998). Veterans were also excluded if they had current or a history of any neurodegenerative disease, active suicidal ideation, concurrent participation in any form of psychotherapy, or prior focused behavioral treatment for HD.

### 2.3. Clinical and self-report assessments

HD symptom severity was assessed using the Saving Inventory-Revised (SI-R) (Frost et al., 2004) as well as the CIR (Frost et al., 2008). The SI-R is a 23-item self-report questionnaire with subscales reflecting the core domains of HD, excessive acquisition, clutter, and difficulty discarding. The CIR is a validated, 9-point, picture-based clutter severity scale. Three rooms of the home (living room, kitchen, and bedroom) are represented via pictures with nine levels of clutter for each room. The total score for the home is generated by averaging individual room scores to reduce differences in the number/type of rooms per home. The CIR was administered to participants in the home and was also completed by the in-home assessor during the home visit. Ratings were done in the same visit, but the assessor did not inform the participant that they were completing the same scale. In addition, Veterans completed the Hoarding Rating Scale-Self Report (HRS-SR) (Nutley et al., 2020; Tolin et al., 2010), a 5-item measure that assesses HD severity. To assess current levels of depression and anxiety symptoms, Veterans completed the Patient-Reported Outcomes Measurement Information System (PROMIS) depression and anxiety scales (Cella et al., 2010; Pilkonis et al., 2011). Functional scales included three self-report measures to assess overall levels of disability (WHO Disability Assessment Schedule 2.0 (WHODAS 2.0) (Konecky et al., 2014; Üstün, 2010)), functioning (Specific Level of Functioning Scale (SLOF) (Schneider and Struening, 1983)) and quality of life (Quality of Life in Neurological Disorders (Neuro-QoL) Positive Affect and Well-Being Short form (Cella et al., 2012)).

### 2.4. Neuropsychological assessment

The neuropsychological assessment focused on measures of executive functioning and included several subtests from the Delis-Kaplan Executive Function System (D-KEFS), including Verbal Fluency (word generation and inhibition/switching), Design Fluency (visual design generation, inhibition/switching), Trails (graphomotor processing speed, attention, task-switching), Color Word Interference Test (verbal processing speed, inhibition/switching), and Tower (abstract reasoning, planning) (Delis et al., 2001). To further assess abstract reasoning and switching, the Wisconsin Card Sorting Test (WCST) was also administered (Test, 1993). All tests’ primary measures were compared to normative samples based on age (and education and gender when such norms were available) and converted to scaled scores or T-scores, with higher scores indicating better performance. Given the variability across test performance in the HD literature and findings from the previous work, we also included error performance across measures when available (Stumpf et al., 2022; van Roessel et al., 2023; Woody et al., 2014).

### 2.5. Analysis

First, we replicated the creation of error scores for the CIR using the method of van Roessel et al. (2022). The composite scores for the self and assessor CIR scores were used to create the CIR-error metric, the objective measure of insight. The self-CIR subtracted from assessor-CIR, divided by assessor-CIR ((assessor-CIR–self-CIR)/assessor-CIR), captures the discrepancy between self-reported clutter in proportion to objective clutter severity. The more positive the CIR-error score, the higher the underreporting of clutter, or the more impaired insight. Means, ranges, and standard deviations were computed for all demographic, clinical, and neuropsychological measures. We examined the relationship between self and assessor CIR scores, first via a paired *t*-test, then via linear regression for factors that may contribute to CIR-error including, age, education, gender, race, depression, anxiety, number and type of comorbidities, and psychotropic medications in addition to self and assessor ratings with CIR-error as the dependent variable. Finally, linear regressions with CIR-error as the dependent variable were conducted for clinical and neuropsychological outcomes. As this was an exploratory analysis of potentially associated variables, we choose not to correct for multiple comparisons, instead reporting effect sizes (Cohen’s *d* with small 0.2, medium 0.5 and large effects 0.8; R^2^ with small 0.01, medium 0.09 and large effect 0.25) and 95 % confidence intervals to assist with any conservative interpretations of associations between insight and potential predictors. Given that missing data were minimal (see differences in n in Table 1) the assumption that data are missing completely at random due to time constraints with at home assessment visits was not felt to add significant risk of bias and all analyses relied on listwise deletion of missing data. Analyses were conducted using SPSS version 29.0.2.0.

## 3. Results

### 3.1. Sample characteristics

A total of 122 Veterans completed in-home baseline assessments, approximately 39 % of whom were female and 57 % were White (see Table 1). The mean age was 62, the mean education level was 15 years, 54 % were on some type of psychotropic medication, and the average participant had two comorbid psychiatric disorders. The most common comorbidity was Major Depressive Disorder (72 %), followed by Other Anxiety Disorder (unclassified or not otherwise specified, 37 %), and Post-Traumatic Stress Disorder (29 %). On the SIHD, 74 % of the sample was rated as having significant acquisition behaviors, and only 10 % (n = 12) of participants were rated as having poor or absent insight. Across measures of HD severity, Veterans at or above clinical cut-off for the SIR (≥42) and had moderate to severe HD symptoms across all other measures. Mean ratings of depression and anxiety symptoms were in the moderate range. Self-reported disability was moderate (WHODAS 2.0), with below average quality of life (Neuro-QOL), and at least some impairment in self-reported functioning (SLOF).

### 3.2. Subjective and objective clutter severity; variability in insight

**Fig. 2.**
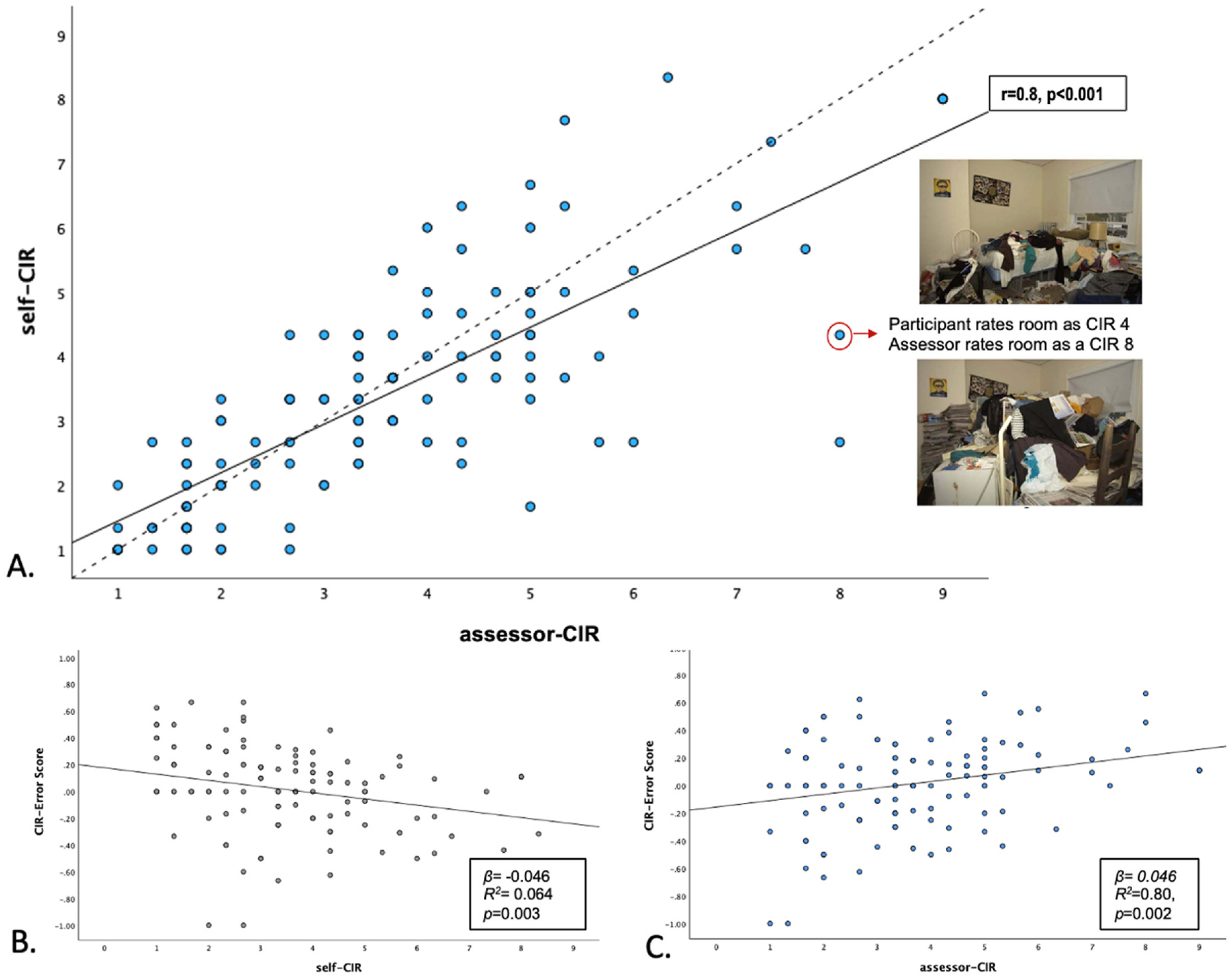
Underreporting of clutter self vs assessor ratings on the Clutter Image Rating Scale A. Assessor (assessor-CIR) and self-rated (self-CIR) scores on the Clutter Image Rating Scale (CIR) are correlated (solid black line) with roughly half of individuals with hoarding disorder (HD) underrating their levels of clutter compared (all points below the dotted line) compared to assessor ratings. The pictures are example of two different ratings on the CIR visual scale which correspond to the circled dot. B. Clutter underreporting is predicted by self-reported clutter with higher self-ratings still being associated with higher objective clutter. C. Clutter underreporting increases with objective clutter score given by an objective assessor (CIR-Error).

Self-assessed CIR scores averaged 3.5, and assessor-rated CIR scores averaged 3.7 (Table 1). Despite a small effect size difference between means, the paired sample *t*-test found self- and assessor-CIR ratings were significantly different from one another (t (121) = −2.1, sd = 1.2, p = 0.018; *d* = 0.19) (Fig. 2). The average CIR-error score was 0.016 (sd = 0.31, range 1.67) with 46.7 % (n = 58) of the Veterans having an error score above 0, indicating some level of underreporting clutter. This percentage is significantly higher than the 10 % of participants identified as having poor or absent insight on the clinician-rated SIHD. However, of the 12 Veterans rated as having poor or absent insight, all had a CIR-error score above 0, reflecting impaired insight. Also, 20.5 % of the sample had no difference in their score from the assessor and 32 % of the sample overreported their level of clutter to some degree.

Next we used univariate linear regression to assess demographic, HD and mood symptom, and functional predictors of CIR-error. First, we regressed both self and assessor scores on CIR-error. Both self and assessor ratings predicted CIR-error, although in opposite directions, with self-ratings having a negative relationship (*β* = −0.046 (se = 0.015), *R*^2^ = 0.064, *p* = 0.003) and assessor ratings having a positive relationship (*β* = 0.046 (se = 0.014), *R*^2^ = 0.080, *p* = 0.002). These results indicate that the degree of objective clutter severity increases for every 0.46 points on the assessor scale and decreases by the same amount on the self-report scale. In multiple regression models with age, education, gender, race, number and type of psychiatric comorbidity, presence of depression and anxiety symptoms, and psychotropic medication use, both self and assessor ratings remained significant predictors of CIR-error (*p* < 0.001) in opposite directions with large effects, with no demographic factors significantly predicting CIR-error (*F* (12, 91) = 37.7, *p* < 0.001, *R*^*2*^ = 0.76).

### 3.3. CIR-error and clinical assessments

**Fig. 3.**
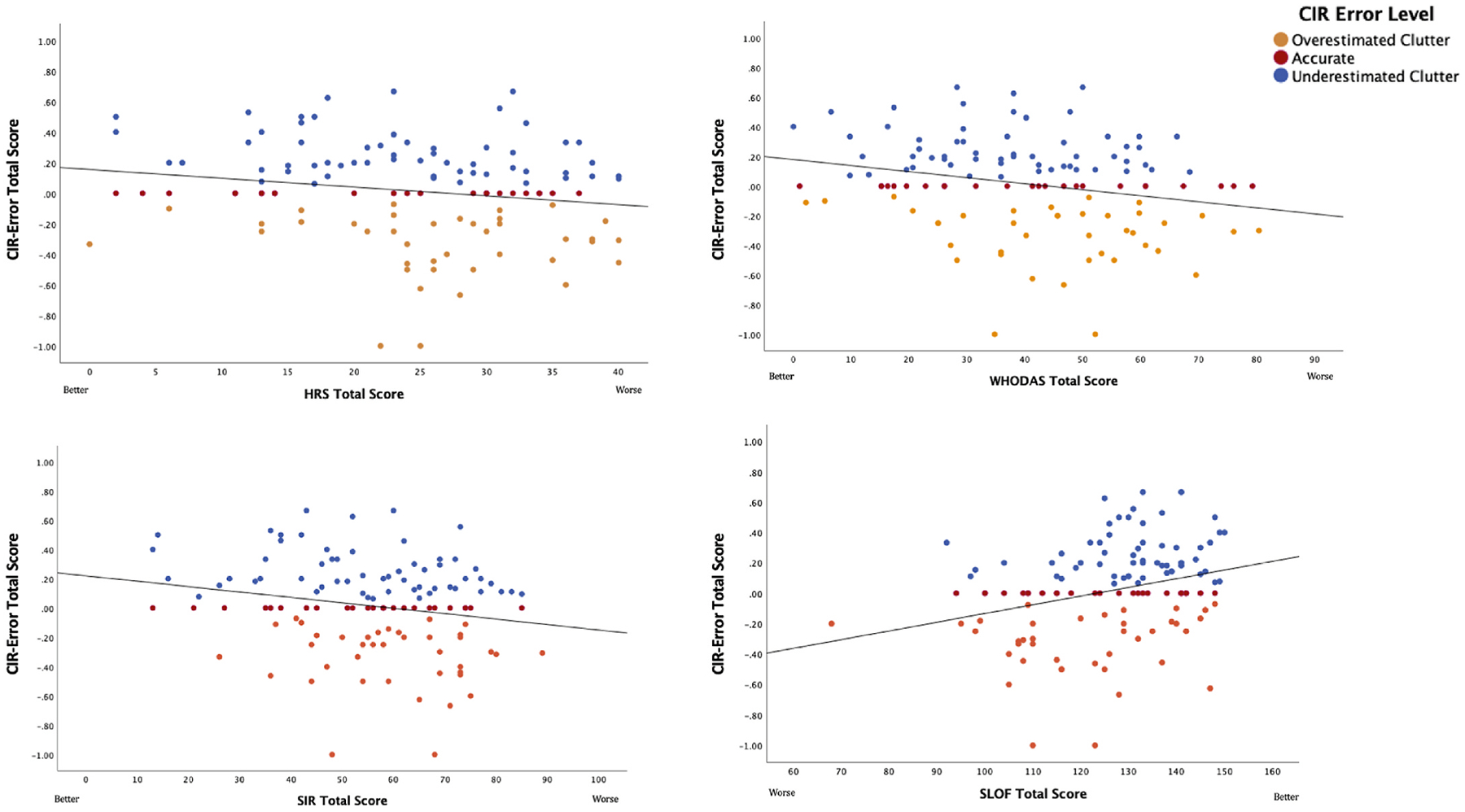
CIR-error score and self-report ratings of HD severity and functioning.

With respect to measures of self-reported HD severity, both the SIR total score and the HRS total score were significantly related to CIR-error scores (SIR total score *β* = −0.004 (se = 0.002), *R*^2^ = 0.041, *p* = 0.026; HRS total score *β* = −0.006 (se = 0.003), *R*^2^ = 0.033, *p* = 0.047). For both measures, lower levels of self-reported HD severity were associated with worse insight (see Fig. 3). Across the SIR subscales (clutter, difficulty discarding, and acquisition), only acquisition was associated with CIR-error (*β* = −0.012 (se = 0.005), *R*^2^ = 0.051, *p* = 0.013), with lower levels of reported acquiring behaviors associated with worse insight. Neither depression nor anxiety symptoms significantly predicted CIR-error. Finally, two of our three measures of functioning were related to CIR-error, both with higher self-reported functioning associated with worse CIR-error (see Fig. 2). The WHODAS 2.0, a self-report measure of disability where higher scores reflect more disability, worse insight was associated with lower levels of self-reported disability (*β* = −0.004 (se = 0.001), *R*^2^ = 0.054, *p* = 0.008). On the SLOF, where a higher score indicates better functioning, worse insight was associated with better self-reported functioning (*β* = 0.006 (se = 0.002), *R*^2^ = 0.084, *p* = 0.001).

### 3.4. CIR-error and neuropsychological performance

A majority of Veterans (n = 91) completed all measures given of the baseline neuropsychological assessment, and all Veterans completed at least some individual tests (Table 1). Across all measures given, Veterans performed in the average range across tests, with a mean score at the midpoint of the standardized scale (scaled score midpoint = 10; T-Score midpoint = 50). Across neuropsychological tests, two were significantly associated with CIR-error (the number of errors on an inhibition/switching test, and the number of errors made on an abstract reasoning test; see Table 2). The two statistically significant results would not have survived a Bonferroni correction and were associated with small effect sizes.

## 4. Discussion

This study is the first to replicate an objective measure of anosognosia or insight in people with HD using the CIR scale completed via self-report and an objective rater. About half of our treatment seeking sample (47 %) had at least some impairment in insight. These findings are similar to previous work and demonstrate that ratings of insight can still be effective when collected simultaneously in the home (van Roessel et al., 2022). Furthermore, Veterans with HD demonstrated similar rates of insight impairment as a prior community sample (van Roessel et al., 2022). The impairments in insight found in our Veteran sample were unrelated to demographic factors, including age, gender, race, and number or type of psychiatric comorbidity. Interestingly, we also found a subgroup of individuals who overrated their clutter; however, we also found no consistent relationships with overreporting and CIR-error score. While prior work has suggested over-reporting may be influenced by motivation or desire to enroll in research (Dimauro et al., 2013; Frost et al., 2008), neither our Veterans nor community members from van Roessel et al. (2022) to be consistent with these factors. In addition, we feel like the use of the specific scale, the CIR, and its visual analog nature may play a factor in the lack of associations with overreporting as compared to global perceptions of hoarding in previous work (Dimauro et al., 2013). Overall, these findings highlight the importance of an objective measure of insight in HD, as well as impaired insight’s relationship to self-reported HD severity, functioning, and cognition.

First, this study underscores the importance of using objective measures to assess anosognosia in Hoarding Disorder (HD) for greater sensitivity to insight impairments. Although all Veterans in our sample were interviewed by trained clinical staff using the SIHD to confirm HD diagnosis—a measure that includes a clinician rating of insight—90 % of Veterans were classified as having good insight, with only 10 % rated as having poor or absent insight. This percentage contrasts sharply with our objective findings, where a substantial proportion of Veterans demonstrated impaired insight. Notably, all 12 individuals rated with poor or absent insight on the SIHD also had impaired CIR-error scores, indicating good clinician specificity. While the SIHD interview may show better sensitivity to impaired insight in non-treatment-seeking samples, as in the initial validation (Nordsletten et al., 2013), our findings highlight the challenges of assessing insight solely through self-report or clinician interviews. These results also emphasize the need for clinicians to anticipate that approximately half of their treatment-seeking HD patients may have at least some degree of impaired insight, even if these deficits are not readily apparent during their initial clinical evaluation.

The impact of impaired insight on self-report measures is evident not only on HD symptom scales but also across various functional measures. Our findings align with van Roessel et al. (2022), showing that lower insight is associated with greater objective clutter severity. Additionally, we observed that lower self-reported HD symptom severity was correlated with higher levels of objective clutter, suggesting a direct interaction between insight and self-reported HD severity. We also found that lower insight was associated with better self-reported functioning on both the WHODAS 2.0 and the SLOF. However, in the absence of objective data on disability or functioning, it remains unclear whether these lower self-reported difficulties genuinely reflect functioning or are another manifestation of anosognosia in HD. In other psychiatric disorders, particularly schizophrenia, better self-reported functioning has consistently been linked to lower insight, a phenomenon often referred to as the insight paradox (Davis et al., 2020). These are the first findings to suggest a similar association between self-reported functioning and lower insight in HD, highlighting the need for further research to determine if this effect is a generalization of the anosognosia or an insight paradox in HD, similar to that seen in schizophrenia. Clinically, our results highlight the importance of using objective data to compare to self-report, even with respect to current functioning. This is particularly applicable for community service agencies who encounter individuals with HD who may be resistant to intervention. Given that distress/impairment is a necessary component for diagnosis, relying on self-report alone, may obscure true impairments due the influence impaired insight on self-reported clutter, symptom severity and functioning. Consideration of treatment goals may need to reflect and address impairments in insight and the potential consequences of better recognition of impaired functioning.

Finally, we examined the relationship between neuropsychological performance and insight. Given that our measures differed from previous work, we conducted a purely exploratory analysis. We found two significant associations with small effect sizes, both related to commission errors on two separate tests. These findings were somewhat consistent with van Roessel et al. (2022), who found that errors on two inhibition-related tasks were associated with impaired insight. However, in our sample, the strongest association was observed on an abstract reasoning measure, the Wisconsin Card Sorting Test (WCST), where fewer errors were linked to better insight. The WCST is a unique task that provides minimal instructions as well as trial-by-trial feedback. It is possible that individuals with low insight in HD have reduced internal sensitivity to error commission, as has been observed in imaging, electrophysiological, and psychophysiological studies (Hough et al., 2016; Mathews et al., 2016; Tolin et al., 2014; Zakrzewski et al., 2018). With external, trial-by-trial feedback, individuals with HD may be better able to adjust their strategy rather than relying on internal performance cues. However, this interpretation is speculative, and further research on error performance and insight in HD is needed.

Despite the importance of these findings, there are several limitations with this work, such as the lack of correction for multiple comparisons due to its exploratory nature. In addition, while this is a replication within a new sample of individuals with HD, Veterans have unique exposures and service experiences that may limit generalization to the overall population. Furthermore, our Veteran sample is on average (62 years), slightly older than typical HD treatment samples (56 years (Rodgers et al., 2021)), and while not present in our dataset, age may be a factor in insight levels. Our study was directly impacted by the onset of the COVID-19 pandemic which resulted in missing data for some measures. In addition, while the external manifestation of clutter in HD allows for quantification of this aspect of the disorder, it is only one aspect. Clutter is not considered the core feature of HD, rather, difficulty discarding is the hallmark (American Psychiatric et al., 2013) and a clutter-focused measure of insight (such as the CIR-error score) may not capture the true severity or nuance of anosognosia in HD. Finally, ours was a treatment-seeking sample and may thus have better insight than non-treatment-seeking samples.

Future research is needed to further establish CIR-Error as an objective measure of insight in HD. Larger samples with more consistent neuropsychological measures, including IQ measures, are essential to better understand the relationship between cognition and insight in HD. Additionally, our finding of better self-reported functioning in Veterans with poorer insight suggests a potential insight paradox in HD, warranting further investigation with objective measures of functioning. Linking biological markers (e.g., neuroimaging, electrophysiology) to insight and neuropsychological performance could provide deeper understanding of the interplay between HD, insight, and error monitoring. These future directions may be especially important to examine in non-treatment seeking samples to better understand how anosognosia functions, not only in HD, but across psychiatric conditions.

## Figures and Tables

**Table 1:** Sample characteristic, symptom & functional measures.

Category	Variable	N	Mean or %	SD	Range
Demographic Characteristics	Age	122	62.1	10.9	26
	Education (Years)	111	15.4	2.4	14
	Gender (Female)	122	38.5 %		
	Race (White)	122	57.4 %		
	Any Psychotropic Medication	108	58 (53.7 %)		
	Antidepressant Medication	108	46 (42.6 %)		
	Other Psychotropic Medication	108	21 (19.4 %)		
Presence of Comorbid Diagnosis	Number of Comorbidities (not including HD)	121	2.3	2.2	9
	Major Depressive Disorder	121	83 (72.2 %)		
	Obsessive Compulsive Disorder	120	10 (8.7 %)		
	Post-Traumatic Stress Disorder	121	33 (28.7 %)		
	Generalized Anxiety Disorder	120	23 (20.3 %)		
	Other anxiety disorder	121	42 (36.5 %)		
Hoarding Measures	Saving Inventory-Revised (SIR)	121	55.8	17.0	76
	SIR Acquiring Subscale	121	14.8	5.9	26
	SIR Clutter Subscale	121	23.3	8.7	35
	SIR Difficulty Discarding Subscale	121	17.7	5.5	25
	Hoarding Rating Scale-Self Report	122	24.4	9.7	40
	Clutter Image Rating Scale-Self	122	3.5	1.8	7.3
	Clutter Image Rating Scale-Assessor	122	3.7	1.9	8.0
	Structured Interview for Hoarding Disorder (SIHD) Insight Specifier	122	12 (9.8 %)		
	SIHD Excessive Acquisition Specifier	122	90 (73.8 %)		
Depression & Anxiety Symptoms	PROMIS Anxiety T-Score	75	60.4	9.4	46
	PROMIS Depression T-Score	75	57.8	9.1	40
Functional Measures	World Health Organization Disability Assessment Scale 2.0	122	40.1	18.4	80
	Specific Level of Functioning Scale	121	126.0	15.7	82
	Neuro-QOL Positive Affect & Wellbeing T-Score	75	48.6	7.6	42

* Other Psychotropic Medications include Antipsychotic, Benzodiazepine, Anti-convulsant and/or Stimulant.

**Table 2:** Neuropsychological performance and associations with CIR-error.

Neuropsychological Test Main Outcome	N	Mean ss	SD	Min	Max	As predictor for CIR-error			
						β	p	R^2^	95 % CI
DKEFS Verbal Fluency: Letter	109	10.4	3.2	4	18	−0.006	0.51	0.004	−0.23; 0.01
DKEFS Verbal Fluency: Category	109	10.8	3.5	2	19	0.004	0.67	0.002	−0.01; 0.02
DKEFS Verbal Fluency: Switching Accuracy	109	9.6	3.4	1	17	0.001	0.95	0.000	−0.02; 0.02
DKEFS Verbal Fluency: Total Repetition Errors	109	10.0	2.6	1	13	−0.023	0.05	0.035	−0.05; 0.00
DKEFS Design Fluency Total Correct	93	10.0	2.8	3	17	−0.008	0.48	0.005	−0.03; 0.01
DKEFS Design Fluency Filled + Empty Total Correct	92	10.2	3.0	4	17	−0.001	0.95	0.000	−0.02; 0.02
DKEFS Design Fluency Switching Total Correct	92	10.4	2.8	1	16	0.007	0.52	0.005	−0.02; 0.03
DKEFS Trails Visual Scanning	95	10.3	2.9	2	15	0.010	0.37	0.009	−0.01; 0.03
DKEFS Trails Number Sequencing	95	11.2	2.8	1	16	0.006	0.56	0.004	−0.02; 0.03
DKEFS Trails Letter Sequencing	95	11.1	2.6	1	15	−0.004	0.75	0.001	−0.03; 0.02
DKEFS Trails Switching	95	10.2	2.9	1	15	0.008	0.47	0.006	−0.01; 0.03
DKEFS Trails Motor Speed	94	10.9	2.4	1	16	0.009	0.52	0.005	−0.02; 0.04
DKEFS Trails Switching Errors	93	10.3	2.5	1	12	0.011	0.40	0.008	−0.01; 0.04
DKEFS Color Word Inhibition	107	9.9	3.1	1	16	0.008	0.40	0.007	−0.01; 0.03
DKEFS Color Word Inhibition Errors	106	10.2	2.9	1	13	0.008	0.48	0.05	−0.01; 0.03
DKEFS Color Word Inhibition/Switching	107	10.0	3.4	1	15	0.003	0.71	0.001	−0.02; 0.02
DKEFS Color Word Inhibition/Switching Errors	106	10.3	3.2	1	13	−0.21	**0.03**	**0.034**	**−0.04; −0.02**
DKEFS Tower Total Achievement	94	10.4	2.9	3	16	0.005	0.64	−0.008	−0.02; 0.03
DKEFS Tower Rule Violations Per Item Ratio	84	9.9	2.9	2	17	−0.007	0.77	0.011	−0.04; 0.01
WCST Categories Completed (Raw Score)	91	3.9	2.2	0	6	0.027	0.06	0.028	0.00; 0.06
WCST Total Errors (T-Score)	91	42.5	11.6	21	80	0.006	**0.03**	**0.040**	**0.00; 0.01**
WCST Total Perseverative Errors (T-Score)	91	44.8	10.5	20	80	0.005	0.09	0.021	0.00; 0.01

Bold indicates *p* values < .0.05; ss = scaled score; WCST= Wisconsin Card Sorting Test; all scores reported in scaled scores unless otherwise specified; average scaled score is 10; average T-score is 50; Total possible correct WCST Categories is 6.
